# Altered gait parameters in distracted walking: a bio-evolutionary and prognostic health perspective on passive listening and active responding during cell phone use

**DOI:** 10.3389/fnint.2023.1135495

**Published:** 2023-11-10

**Authors:** Hassan Bazzi, Anthony T. Cacace

**Affiliations:** ^1^Department of Biological Sciences, Wayne State University, Detroit, MI, United States; ^2^Department of Communication Sciences and Disorders, Wayne State University, Detroit, MI, United States

**Keywords:** gait, GAITRite^®^ Walkway System, evolutionary biology, bipedal locomotion, cell phone

## Abstract

The underpinnings of bipedal gait are reviewed from an evolutionary biology and prognostic health perspective to better understand issues and concerns related to cell phone use during ambulation and under conditions of distraction and interference. We also consider gait-related health issues associated with the fear of or risk of falling and include prognostic dimensions associated with cognitive decline, dementia, and mortality. Data were acquired on 21 healthy young adults without hearing loss, vestibular, balance, otological or neurological dysfunction using a computerized walkway (GAITRite^®^ Walkway System) combined with specialized software algorithms to extract gait parameters. Four experimental conditions and seven temporo-spatial gait parameters were studied: gait velocity, cadence, stride length, ambulatory time, single-support time, double-support time, and step count. Significant main effects were observed for ambulation time, velocity, stride velocity, and double-support time. The greatest impact of distraction and interference occurred during the texting condition, although other significant effects occurred when participants were verbally responding to queries and passively listening to a story. These experimental observations show that relatively simple distraction and interference tasks implemented through the auditory sensory modality can induce significant perturbations in gait while individuals were ambulating and using a cell phone. Herein, emphasis is placed on the use of quantifiable gait parameters in medical, psychological, and audiological examinations to serve as a foundation for identifying and potentially averting gait-related disturbances.

## 1. Introduction

One of the most remarkable achievements of human evolution was the transition from *quadrupedal-to-bipedal* gait; a phenomenon that changed an “arboreal” existence to a “terrestrial” lifestyle, whereby early man (Hominids: genus, *Homo*; species, *sapiens*), assumed an upright posture and where land-based bipedal gait became the primary mode-of-ambulation. Available evidence suggests that genetic adaptations, anatomical/physiological, biomechanical, and cognitive underpinnings contributed to this transition (Eccles, 1989; Zehr and Duysens, 2004; Gramsbergen, 2005; Hicks and Onodera, 2012; Gruss and Schmitt, 2015; Wilson et al., 2019; Guillaud et al., 2020; Nóbrega-Sousa et al., 2020). A three-tiered model consisting of supraspinal influences, spinal cord pattern generators from postural and motor reflexes in ascending and descending brainstem pathways, and feedback mechanisms associated with multisensory and motor-integration systems directly influenced the regulation and maintenance of normal gait and locomotion (Zehr and Duysens, 2004). Computer simulations and models of locomotion incorporating entrainment between musculoskeletal, neural systems and the environment have also been proposed (Taga, 1995). These intrinsic multidimensional considerations set the stage for asking the highly relevant question; “why is the study of gait important?”

Addressing this enquiry provides a unique opportunity to examine a host of issues related to evolutionary biology, health-related factors associated with the risk or fear of falling, prognostic indicators of cognitive decline, dementia, and mortality, as well as other factors related to the susceptibility of gait parameters to distraction and interference effects (e.g., Hausdorff et al., 2000; Verghese et al., 2007; Studinski et al., 2011; Fattal et al., 2018).

From an evolutionary biology perspective, benefits that have emerged from the transition to a two-legged stance includes the adeptness of individuals to scavenge for food from low-hanging tree branches and bushes and the improved ability to survey the surrounding environment for drinkable water. Both elements are essential for sustaining life, promoting growth, and preserving the viability of the species. Other advantages include carrying objects and transporting offspring over long distances, across various terrains and through diverse aquatic environments. Bipedal deportment also contributed toward enhancing manual dexterity by freeing up the arms, hands, and fingers to fabricate tools for constructing shelters, assembling armaments for hunting, developing armaments for protection, and in appropriating the use of fire for cooking, providing light to the immediate environment, and maintaining warmth (Hewes, 1964; Clark and Harris, 1985; Brain and Sillent, 1988). As theorized, the success of these various behaviors served to increase the chances of self-preservation, survival, and overall reproductive abilities through natural selection (Darwin, 1895).

The sophistication and adaptive nature of early hominids grew as their language proficiency increased. This effect draws on the intimate linkage between interactive communication abilities and contributions made through the growth of culture (Castro et al., 2004; Smith and Kirby, 2008; Chiu, 2011). Indeed, “cultural enrichment” was the cornerstone for transmitting knowledge from one generation to the next where heritable adaptations^1^ and cognitive systems of learned behaviors were given a high priority for this emerging enterprise to expand and flourish (Lachlan and Feldman, 2003).

Among the various areas that address the importance of studying gait, “risk of, or fear-of-falling” is a primary exemplar (e.g., Houry et al., 2016; Gazibara et al., 2017; Schniepp et al., 2017; Lavedán et al., 2018). In older adults, fear-of-falling is a distinct concern particularly when we take into account those individuals that have experienced physical setbacks from chronic health conditions such as strokes and metabolic disease like diabetes (e.g., D’Silva et al., 2016; Goh et al., 2016; Gazibara et al., 2017; Lavedán et al., 2018; Hewston and Deshpande, 2018). The Centers for Disease Control and Prevention (2015) recognizes that falling is a major health concern, where the financial impact and economic burden is extraordinarily high (∼$50 billion annually). The cost associated with falls is attributed in large part to medical complications from broken hip bones, prolonged hospitalizations, and even death (e.g., James et al., 2017; Florence et al., 2018; Cao et al., 2021). Along with the direct consequences of a fall, the loss of independence is a distinct post fall co-morbidity where individuals adopt compensatory strategies such as becoming more sedentary and ambulating more cautiously as a way to reduce or avoid unwanted vestibular and balance-related symptoms (Guinand et al., 2012; Sun et al., 2014).

A notable clinical area leading to the risk-of-falling is associated with bilateral vestibulopathies (BVPs); a complex condition characterized by symptoms of imbalance, chronic disequilibrium, postural instability, dizziness, disabling vertigo, difficulty walking in a straight line, and oscillopsia. In oscillopsia, this condition can be particularly disconcerting because the oculomotor system is unable hold images on the retina in a stable manner causing stationary objects in the visual field to be perceived as jumping, jiggling, or oscillating (Kim et al., 2011; Strupp et al., 2017; Hermann et al., 2018; Lucieer et al., 2020; Kim and Kim, 2022).

In addition, there is evidence indicating that specific temporo-spatial gait parameters have prognostic value in identifying individuals experiencing “cognitive decline” (e.g., Verghese et al., 2007; Taniguchi et al., 2012; Mielke et al., 2013; Anderson-Mooney et al., 2016; Morris et al., 2017; Savica et al., 2017; Valkanova and Ebmeier, 2017; Li et al., 2018; Dobbels et al., 2019, 2020; D’Silva et al., 2022). A specific signature of abnormal gait has been shown for *double-support time;* a specific gait parameter associated with Alzheimer’s disease and Lewy body dementia (McArdle et al., 2019). Not surprisingly, other neurological conditions such as Parkinson’s disease (Mirelman et al., 2019), Huntington’s disease (Koller and Trimble, 1985), Amyotrophic Lateral Sclerosis (Hausdorff et al., 2000; Xia et al., 2016), and Multiple Sclerosis (MS) (Postigo-Alonso et al., 2018) also have gait-related impairments. Of particular interest is the highly provocative observation that loss of gait speed (>10 cm/s) is associated with increased mortality (Wilson et al., 2002; Hardy et al., 2007; Jahn et al., 2010; Fattal et al., 2018).

When we consider all of these factors as a whole, the importance of solidifying how people communicate and maintain their social beliefs and values represents important characteristics associated with the evolution of gait. In contemporary society, electronic media influenced these interactions through intermediaries such as radio, television, and the Internet. However, it was the advent and proliferation of mobile cellular devices (cell phones) that was the primary contributor toward enhancing personal communication and advancing culture. Cell phone usage freed landline communication systems from their wired tether and set the stage for the prominent role it plays in all aspects of societal life worldwide. However, the dependence on cell phone use did not come without a cost. Maladaptive behaviors and safety concerns emerged when individuals were walking, talking, and texting. These maladaptive behaviors gave rise to increased accidents, injuries to the body, unintended emergency-room visits, and even death (Schwebel et al., 2012; Nasar and Troyer, 2013; Smith et al., 2013; Thompson et al., 2013; Parr et al., 2014; Schabrun et al., 2014; Licence et al., 2015). Therefore, issues related to the effects of distraction and interference on cell phone use while ambulating represents a tour de force contributor toward addressing the question of why the study of gait is important.

Therefore, the experimental objectives we propose will examine which of seven common temporo-spatial gait parameters are susceptible to distraction and interference effects and evaluate how this objective can aid in the early identification and codification of medical, psychological, psychiatric, and/or neurological dysfunctions.

## 2. Materials and methods

### 2.1. Participants

Twenty-one adults ranging in age from 21 to 31 years (11 males; mean age 24.4 years, standard deviation, 2.6 years; 10 females, mean age 25.4 years, standard deviation, 0.97 years), served as participants in this experiment. Inclusion criteria required a negative history of vestibular, balance and hearing-related dysfunctions including the presence of active otological and neurological disease. Exclusion criteria included hearing loss ≥25 dB HL at octave frequencies from 0.5, 1, 2, and 4 kHz, documented via a hearing screening test performed at 20 dB HL bilaterally. Also excluded were those individuals with a history of concussion, high-level noise exposure or blast overpressures from occupational, recreational, or military experiences. Lastly, because recruitment of participants was based on non-probabilistic sampling, the acquisition of subjects constituted a convenience sample, whereby friends, relatives, and university students were invited to participate. These factors and conditions satisfied the criteria for approval of this investigation from the Wayne State University Institutional Review Board (IRB).

### 2.2. Experimental design

**FIGURE 1:**
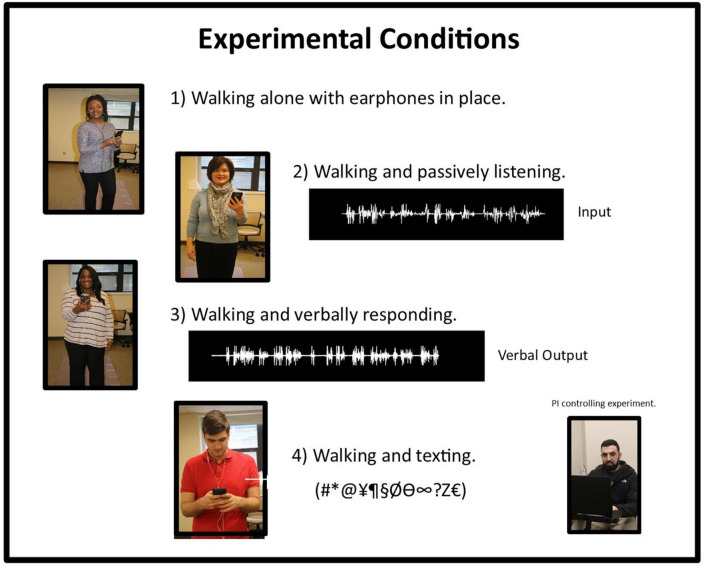
Graphic illustration representing the four conditions used in this experiment. Under Condition 2, the waveform represents the input to the insert earphone. Under condition 3, the waveform represents the verbal output of the response of the individual to a query. The bottom right-hand photograph shows the PI controlling the experiment with a laptop computer.

All testing was performed with eyes open and with earphones in place bilaterally. Acoustic activations were elicited from insert earphones connected to a single cell phone (Samsung, Galaxy S8), since determining whether ear-of-stimulation played a role in altering gait parameters was a distinct component of this experimental design. Four experimental conditions were applied, where each task increased in complexity from the previous condition. The experimental tasks studied, included: (1) walking and holding a cell phone, with earphones in place but without any acoustic stimulation being applied, (2) walking while listening to a pre-recorded passage (story) presented separately to left and right ears, (3) walking while listening to a pre-recorded passage presented separately to left and right ears and answering (responding to) questions, and (4) walking while listening to a recorded text passage and responding to questions via text messaging. In all instances, acoustic input was presented separately to left and right ears. Figure 1 provides an illustration of these experimental conditions.

### 2.3. Instrumental and statistical analyses

The walking tasks were performed using the GAITRite^®^ Walkway System (CIR Systems, Inc., Franklin, NJ, USA), which is a carpeted runner with sensors embedded within the fabric of the walkway. When fully extended on a flat surface, the carpeted walkway is 16 feet in length and 2 feet 10 inches in width. The walkway was connected to a laptop computer (Lenovo, Ideapad, model: 310) via a universal serial bus (USB) and gait data were collected by specialized acquisition software, designed, and provided by the manufacturer.

Data from individual runs were stored in coded, de-identified computer files and subsequently transferred to an external hard drive (Western Digital) for back-up. All testing was performed in a well-lit, temperature controlled quiet environment, designated for student experimentation. This testing environment was held constant without any spatial modifications or undue distractions between different days of testing. During the assessment trials, participants were instructed to walk at a comfortable pace, normal for their ambulatory disposition. Neither a metronome nor other type of external timing device was utilized to control or maintain speed-of-walking.

Participants walked back and forth on the carpeted runner 4 times, totaling 64 feet of distance traveled. This ambulation-based travel dimension was chosen with the intent of increasing sample size of the gait parameters to be studied. The pre-recorded verbal material was presented via insert earphones at a comfortable-listening level, which approximated 75 dB sound-pressure level (SPL). Quantitative acoustic measurements verifying the acoustic output from the earphones were made via a Zwiskocki coupler (Zwislocki, 1980; model DB 100), attached to a sound-level meter (Bruel & Kjaer, model 2209), with a 1/2” condenser microphone (Bruel & Kjaer, model 3134). Plumber’s putty secured the earphone in place and provided an acoustic seal to the coupler during the acoustic calibration process.

Seven common temporo-spatial gait parameters were extracted for study. These included gait velocity, cadence, stride length, ambulatory time, single-support time, double-support time, and step count. Definitions of these various gait parameters were adapted from the User’s Manual of the device (GAITRite, 2013) and are provided in Table 1. Our approach to this topic followed a model endorsed by Lord et al. (2013), which was used to map gait into five general domains covering pace, rhythm, variability, asymmetry, and postural control.

**TABLE 1:** Definitions of spatiotemporal gait parameters.

Gait parameter	Definition
Ambulation time	Represents the time in seconds (s) to complete the entire recording epoch.
Velocity	The rate-of-change over time, which is obtained after dividing the distance traveled by the ambulation time. It is expressed in centimeters-per-second (cm/s).
Stride velocity	The rate-of-change over time for individual strides, averaged across the entire recording epoch.
Cadence	Steps per minute, as a measure of intensity.
Stride length	The line of progression between the heel points of two consecutive footprints of the same foot (left to left, right to right). The unit of measure in centimeters.
Ambulatory time	The time elapsed between first contact of the first and the last footfalls; measured in seconds (s).
Double-support time	The two periods when both feet are on the floor, are called initial double support and terminal double support. Initial double support occurs from heel contact of one footfall to toe-off of the opposite footfall. It is measured in seconds (s) and also expressed as a percent of the Gait Cycle time for the same foot. DS1 is the Initial Double Support for the right foot, while the DS2 is the Initial Double Support for the left foot.
Step count	Number of steps per minute.

Left column shows the gait parameter; right column provides the definition.

### 2.4. Normalization procedures

Because individuals differed in height, leg length, step length, etc., normalization procedures were used to account for individual anthropomorphic variations, such that relevant comparisons can be made during statistical analyses (Hof, 1996; GAITRite, 2013). These normalization procedures are depicted below with additional information provided in Footnote 2:^2^

$$\mathrm{Normalized}\,\mathrm{velocity}=\frac{\text{Velocity}}{\sqrt{\text{D}}\times \text{g}}$$

$$\mathrm{Normalized}\,\mathrm{step}\,\mathrm{length}=\frac{\mathrm{Step}\,\mathrm{length}}{\text{D}}$$

$$\text{Normalizedcadence}=\frac{\sqrt{\text{D}}}{\text{g}}$$

### 2.5. Statistical analyses

For each of the gait parameters studied, separate 2 × 4 repeated measures analyses of variance (ANOVAs) were used to evaluate the effects of ear-of-stimulation (left vs. right) and condition (four experimental tasks). If main effects were significant and occurred without any statistical interactions, then *post hoc* analyses were performed using the Tukey Honestly Significant Difference Test (HSDT) to further clarify the statistical outcomes of all pairwise group-mean comparisons. In addition, a separate Pearson’s product-moment correlation analysis was performed to evaluate relationships among all of the 16 temporo-spatial gait parameters studied.

## 3. Results

All participants completed the study in its entirety. There were no missing values, adverse reactions, or negative events reported by any of the individuals, including ear pain attributed to insert earphone usage, headaches, stumbling, falling, or mental distress. Based on the descriptive statistics and findings from the ANOVAs, significant effects were observed for ambulation time, velocity, stride velocity, single and double-support times. In terms of their overall effects, each of the four conditions were ordered in the following manner: texting, responding verbally to queries, and passive listening.

**FIGURE 2:**
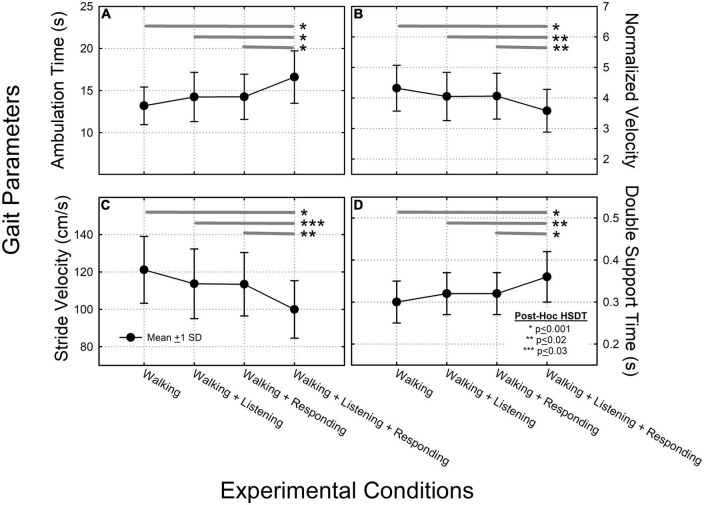
Data summarizing the ANOVA results across each of the four experimental conditions, for: **(A)** ambulation time; **(B)** normalized velocity; **(C)** stride velocity; and **(D)** double-support time. In each of these four plots, filled circles connected by solid lines and error bars characterize group mean values, ±1 standard deviation (SD). Thick dark gray horizontal lines represent results from the Tukey *post-hoc* Honestly Significant Difference (HSD) test where significant pair-wise group mean comparisons are characterized by asterisks depicting: *HSD < 0.001, **HSD < 0.002, ***HSD < 0.003.

Descriptive statistics of the gait variables studied are provided in Table 2. Statistical results from the ANOVAs are outlined below and are also graphed as line plots (Figures 2A–D). The individual line plots show experimental results where the different experimental conditions are shown on the *x-axis* and specific gait metrics are provided on the *y-axis*. *Post hoc* comparisons for each condition are also depicted on each plot.

**TABLE 2:** Descriptive statistics for the temporo-spatial gait parameters for left ear (LE) and right ear (RE), respectively.

Variable	Descriptive statistics					
	**Ear**	**Sample size**	**Mean**	**SD**	**Minimum value**	**Maximum value**
Ambulation time	Left	21	16.85	12.03	25.22	3.53
Velocity	Left	21	98.27	66.70	121.70	15.79
Step count	Left	21	26.52	20.00	33.00	3.54
Cadence	Left	21	96.10	75.20	110.40	10.52
Stride length	Left	21	122.61	102.11	144.98	11.09
Stride velocity	Left	21	98.73	67.43	120.96	15.69
Single-support time	Left	21	0.45	0.40	0.57	0.06
Double-support time	Left	21	0.36	0.27	0.51	0.06
Ambulation time	Right	21	16.35	10.99	21.28	2.72
Velocity	Right	21	100.71	74.30	129.40	15.25
Step count	Right	21	26.24	20.00	31.00	3.16
Cadence	Right	21	97.44	77.00	109.20	10.25
Stride length	Right	21	123.82	106.70	145.44	10.64
Stride velocity	Right	21	101.12	74.88	130.54	15.32
Single-support time	Right	21	0.45	0.40	0.55	0.05
Double-support time	Right	21	0.35	0.27	0.47	0.05

### 3.1. Ambulation time

A significant main effect of ambulation time (*F* = 11.35, *p* < 0.001) but not ear-of-stimulation was observed (*F* = 1.66, *p* > 0.05). The two-way ear-of-stimulation × ambulation-time interaction was not significant (*F* = 0.28, *p* > 0.05). When data were collapsed across ear-of-stimulation, systematic increases in ambulation time were observed across conditions. The *post hoc* HSDT showed significant differences among conditions 1 vs. 4 (*p* < 0.001), 2 vs. 4 (*p* < 0.001), and 3 vs. 4 (*p* < 0.001). Under these conditions, *texting* had the greatest impact on increasing ambulation time (Figure 2A).

### 3.2. Normalized velocity

There was a significant main effect of velocity (*F* = 6.96, *p* < 0.001) but not ear-of-stimulation (*F* = 0.58, *p* > 0.05). The two-way velocity × ear-of-stimulation interaction was not significant (*F* = 0.06, *p* > 0.05). When data were collapsed across ear-of-stimulation, velocity systematically decreased across conditions. The *post hoc* HSDT showed significant differences among conditions 1 vs. 4 (*p* < 0.001), 2 vs. 4 (*p* < 0.02), and 3 vs. 4 (*p* < 0.02) (Figure 2B).

### 3.3. Stride velocity

There was a significant main effect of stride velocity (*F* = 10.76, *p* < 0.001) but not ear-of-stimulation (*F* = 0.88, *p* > 0.05). The two-way stride velocity × ear of stimulation interaction was not significant (*F* = 0.05, *p* > 0.05). When data were collapsed across ear-of-stimulation, systematic decreases in stride velocity were observed across conditions. The *post hoc* HSDT showed significant differences among conditions 1 vs. 4 (*p* < 0.001), 2 vs. 4 (*p* < 0.003), and 3 vs. 4 (*p* < 0.002) (Figure 2C).

### 3.4. Double-support time

There was a significant main effect on double-support time (*F* = 10.55, *p* < 0.001) but not ear-of-stimulation (*F* = 1.02, *p* > 0.05). The double-support time by ear-of-stimulation interaction was not significant (*F* = 0.04, *p* > 0.05). When data were collapsed across ear-of-stimulation, *post hoc* HSDT showed significant differences between conditions 1 vs. 4 (*p* < 0.001), 2 vs. 4 (*p* < 0.002), and 3 vs. 4 (*p* < 0.001) (Figure 2D).

### 3.5. Correlation matrix

**FIGURE 3:**
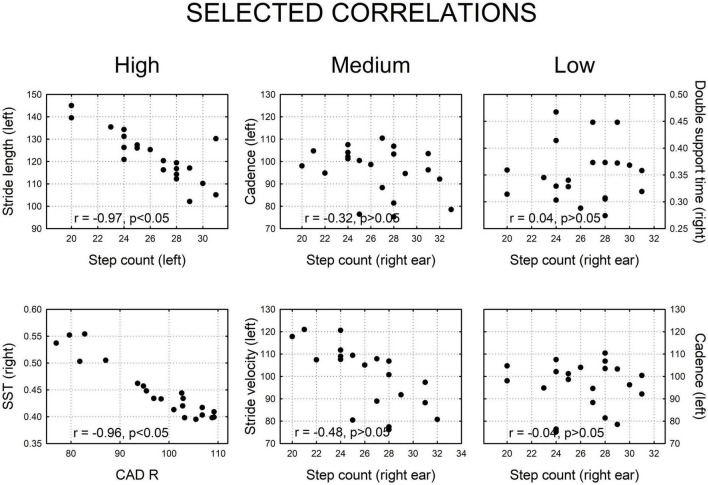
Selected examples of two dimensional correlation plots showing high, medium, and low correlations, derived from this figure. Strong correlations were statistically significant (*p* < 0.05); medium and low correlations were not significant (*p* > 0.05).

Because the texting condition had the most prominent effect, the resultant correlation matrix showed that the majority [96 of 120 pairwise temporo-spatial gait parameters, (80%)] were significantly correlated (Table 3), where significant *r* values are shown in bolded print (*p* ≤ 0.05). We also show examples of scatter plots of strong, medium, and low correlations (Figure 3).

**TABLE 3:** Pearson product-moment correlation matrix for 16 temporospatial gait parameters for LE and RE, respectively.

VARIABLES	AT L	VEL L	SC L	CAD L	SL L	SV L	SST L	DST L	AT R	VEL R	SC R	CAD R	SL R	SV R	SST R	DST R
AT (LE)	1.00															
VEL (LE)	**−0.98**	1.00														
SC (LE)	**0.82**	**−0.76**	1.00													
CAD (LE)	**−0.80**	**0.84**	−0.32	1.00												
SL (LE)	**−0.81**	**0.78**	**−0.97**	0.32	1.00											
SV (LE)	**−0.98**	**1.00**	**−0.75**	**0.85**	**0.77**	1.00										
SST (LE)	**0.69**	**−0.76**	0.20	**−0.93**	−0.24	**−0.76**	1.00									
DST (LE)	**0.79**	**−0.78**	0.41	**−0.88**	−0.37	**−0.79**	**0.68**	1.00								
AT (RE)	**0.89**	**−0.91**	**0.77**	**−0.69**	**−0.80**	**−0.91**	**0.65**	**0.61**	1.00							
VEL (RE)	**−0.94**	**0.98**	**−0.74**	**0.81**	**0.78**	**0.98**	**−0.76**	**−0.72**	**−0.94**	1.00						
SC (RE)	**0.51**	**−0.49**	**0.80**	−0.04	**−0.81**	**−0.48**	0.01	0.05	**0.73**	**−0.53**	1.00					
CAD (RE)	**−0.76**	**0.83**	−0.29	**0.98**	0.32	**0.84**	**−0.94**	**−0.84**	**−0.72**	**0.83**	−0.06	1.00				
SL (RE)	**−0.75**	**0.73**	**−0.95**	0.25	**0.99**	**0.72**	−0.20	−0.28	**−0.79**	**0.75**	**−0.86**	0.27	1.00			
SV (RE)	**−0.94**	**0.98**	**−0.74**	**0.81**	**0.77**	**0.98**	**−0.76**	**−0.72**	**−0.94**	**1.00**	**−0.53**	**0.83**	**0.75**	1.00		
SST (RE)	**0.71**	**−0.78**	0.25	**−0.93**	−0.29	**−0.79**	**0.99**	**0.70**	**0.70**	**−0.80**	0.07	**−0.95**	−0.25	**−0.80**	1.00	
DST (RE)	**0.71**	**−0.73**	0.34	**−0.84**	−0.31	**−0.74**	**0.66**	**0.96**	**0.59**	**−0.72**	0.04	**−0.84**	−0.26	**−0.72**	**0.68**	1.00

Bold values represent significant correlations (*p* < 0.05).

AT, ambulation time; Vel, velocity; SC, step count; CAD, cadence; SL, stride length; SV, stride velocity; SST, single-support time; DST, double-support time.

## 4. Discussion

Alterations in various gait parameters were significantly affected by perceptual, motor, and cognitive distraction/interference effects while individuals were ambulating and using a cell phone. The consistency of these data is in accordance with excellent test-retest reliability of the GAITRite^®^ Walkway System (see Menz et al., 2004). We show that the greatest impact of distraction and interference occurred during the texting condition, where Ambulation Time and Double-Support time *increased* and where Velocity and Stride Velocity *decreased*. When individuals were passively listening to a story or engaged in verbally responding to queries, gait parameters were also altered.

Other researchers have described comparable findings. For example, Licence et al. (2015) used similar but not identical experimental conditions to evaluate gait-related interference effects. They studied normal walking, texting + walking, and texting + walking while listening to a cognitively distracting task. Licence et al. (2015) analyzed gait features using a three-dimensional optical-motion-analysis system (Qualsys, Sweden). In contrast to the GAITRite^®^ Walkway, the optical motion system offered added flexibility in terms of applying more complex walking paths and allowing for the use of small barriers in these walking paths which subjects had to negotiate. In their assessment, gait parameters included the overall time to complete the obstacle course, obstacle clearance height, step frequency, step size, double support phase, and lateral deviation. Their results indicated that participants needed greater time to complete the course particularly while texting and during distraction. Step frequency, step size, and double support phase all increased significantly. Together, these effects were interpreted as contributing to a more “cautious” gait-related walking pattern; a comparable result reported by Russo et al. (2018). Thus, when competing for cognitive resources and attentional demands, or when motor processes are engaged, peripheral receptors, shared brain areas and neural circuits in the central nervous system can be disrupted by these events.

### 4.1. Ear-of-stimulation

We also found that ear-of-stimulation did not significantly alter gait parameters when individuals were ambulating and using a cell phone. This result implies that models or theories of hemispheric specialization, as proposed by many prominent scientists (i.e., Gazzaniga, Sperry, Chomsky, Hugdahl, Hickok, Poeppel, and Pinker) are unrelated to the effects we observed (see Corballis, 2015 for a comprehensive review).

### 4.2. Verbal communicative interactions while ambulating

While there is a modest literature on gait-related dual-task interference paradigms (Bender et al., 1997; Ghai et al., 2017), there is an *absence* of experimental data on gait-related disturbances occurring when individuals are simultaneously conversing with nearby companions, partners, or friends during cell phone use. This distinction is in contrast to the area of distracted driving where driver-passenger interactions has been studied extensively. Driving while eating, smoking, listening to music, and viewing objects or scenes in the external environment are examples of alternative distraction and interference effects worth noting (e.g., Michon, 1985; Cohen et al., 2003; Stavrinos et al., 2017; Theofilatos et al., 2018; Regan and Oviedo-Trespalacios, 2022).

### 4.3. Distinct neurological and cognitive co-morbidities of auditory distraction and interference while ambulating: texting

In the current study, it was found that texting while ambulating had the greatest impact on ambulatory time, velocity, stride velocity, and double-support time. While these effects are consistent with other reports in the literature (Lamberg and Muratori, 2012; Schwebel et al., 2012; Schabrun et al., 2014; Agostini et al., 2015), they were unique since they were limited to auditory-specific tasks.

Schabrun et al. (2014) found that while reading text and when text messaging while ambulating, a noticeable impact on gait was observed. These effects were manifested by individuals walking at slower speeds, having a greater deviation from walking in a straight path, and having increased lateral-step strides. Interestingly, these authors also evaluated changes in head motion where increased *rotation* and reduced relative motion of the head were observed. Schwebel et al. (2012) and Lamberg and Muratori (2012) used virtual reality environments to assess an individual’s ambulatory performance while texting and crossing the street. It was noted that when individuals were engaged in these behaviors, they tended to look away more frequently from the street environment than those who were not distracted by texting. They also experienced more simulated hits by motor vehicles.

Agostini et al. (2015) asked the intriguing question, “Does texting while walking really affect gait in young adults?” In their experimental conditions (walking and texting) over a 15-m path and for a duration of 3 min, only small modifications characterized by a reduction in gait speed were observed. Interestingly, when kinemetric analysis was used to evaluate changes in ankle and knee motions, these anatomical structures were *not* overtly affected by texting. However, some distinct muscle groups did show effects. For example, there was delayed onset activation of the left gastrocnemius lateralis muscle and increased co-contraction of the tibialis anterior and gastrocnemius lateralis muscles. As noted by Cibulka et al. (2017), this observation could be an important consideration because dorsiflexion of the tibialis anterior muscle is critical for clearing the foot off the ground. In retrospect, it appears that very little is known regarding gastrocnemius lateralis muscle contraction and its impact on ambulation particularly with respect to different foot and stance positions. Thus, the evolution of gait related musculoskeletal interactions remains to be fully elucidated.

### 4.4. Gait velocity

Defined as the rate-of-change of movement as one advances through space, gait velocity is another dimension that has been impacted. In their study, Holtzer et al. (2006) used factor analysis of neuropsychological test scores from cognitively normal elders (*n* = 186) to examine the relationship between cognition and gait velocity under conditions of ambulation alone and ambulation plus interference. These authors found that Verbal IQ, Speed/Executive attention, and memory were significant predictors of gait velocity. While subsequent regression analysis found that all three factors predicted gait velocity without any interference effects, only Speed/Executive Attention and Memory factors but not Verbal IQ predicted gait velocity were affected in the interference conditions. These data suggest that gait velocity and cognitive function have both shared and independent brain substrates contributing to this outcome.

As part of a multivariate study, Hardy et al. (2007) set out to estimate the relationship between 1-year improvement in measures of health and physical function in relation to an 8-year survival period. They evaluated six areas: (1) gait speed, (2) the Short Physical Performance Battery (SPPB), (3) the 36-item Short-Form Health Survey Physical Function Index (F-36 PFI), (4) Global Health, (5) EuroQol, a widely used questionnaire which evaluates quality-of-life in Europe, and (6) the National Health Interview Survey and Activities of Daily Living (NHIS ADL). They found that improvement in gait speed at 1 year was significantly associated with a *reduction* in mortality through the subsequent 8 years-of-life. This effect was associated with a 58% reduction in relative risk and a 17.7% reduction in absolute risk-of-death.

### 4.5. Double-support time

Double-support time characterizes the time during two ambulatory periods when both feet are touching the ground. Postigo-Alonso et al. (2018) found that individuals with MS were particularly vulnerable to cognitive and motor interference effects with double-support time being the most sensitive gait variable involved. Interestingly, another motor variable, “verbal fluency,” proved to be sensitive and specific to cognitive motor interference effects in MS.

### 4.6. Effects of hearing loss

While none of the participants in the current cohort experienced hearing loss over the frequency range studied (0.5–4 kHz), hearing loss *per se* does appear to be a factor which can also alter gait. For example, Szeto et al. (2021) found that decreased pure tone hearing sensitivity (i.e., greater hearing loss) was associated with increased variability in double-support time values. While the precise explanation for this effect remains to be elucidated, it was noted that poor interlimb coordination, deterioration of balance-control mechanisms, and mobility limitations with increased age were possible contributing factors (Gabell and Nayak, 1984; Serrient et al., 2000; James et al., 2017). Szeto et al. (2021) also found that variability associated with double-support time was asymmetric, particularly if/when hearing loss was greater in the right vs. left ear.

Li et al. (2013) analyzed data from the National Health and Nutritional Examination Survey circa 1999–2002 where 1,180 participants, ages 50–69 years, underwent hearing testing and gait assessment. Based on logistic regression analysis using a model that adjusted for demographic and cardiovascular risk factors, Li et al. (2013) found that greater hearing loss was associated with slower gait speed. This effect was independent of all other factors that were studied. Moreover, in a series of studies, Viljanen et al. (2009) studied the relation between falling, fear of falling, and increased hearing loss in older women. They found that older women with poor hearing sensitivity had a higher risk for falls than those with good hearing.

While only a few studies have investigated the relationship between hearing sensitivity and postural control, little is known about the effect of hearing aid use or other assistive auditory devices used by elderly individuals with hearing loss. In this context, Shayman et al. (2017, 2018) found that gait velocity improved with the use of bilateral hearing aids or cochlear implants. In fact, they suggest that cochlear implants could be recognized as “balance implants” and speculate that improvements in gait are attributed to enhanced spatial cues in those cases with severe-to-profound hearing loss.

Berge et al. (2019) investigated the relationship between hearing sensitivity, gait, and postural stability in elderly adults with hearing loss. They compared performances on hard and soft (foam) surfaces, including conditions with eyes open and eyes closed, and with hearing aids turned on and off. Of the dependent variables studied, it was found that hearing aids turned on improved balance function by reducing the standard deviation of velocity. To further reinforce this view, Cornwell et al. (2020) point out the importance of auditory cues in in terms of providing feedback and temporal cues from sounds such as footsteps and other external reference sounds might contribute toward improved ambulation abilities.

## 5. Conclusion

The experimental findings described herein show that relatively simple distraction/interference tasks implemented through the auditory sensory modality can significantly alter temporo-spatial gait parameters while individuals are ambulating and using a cell phone. By considering the timeline of these events dating back to when Hominids transitioned from quadrupedal-to-bipedal gait, we provide a comprehensive overview of this topic from a unique historical perspective. Important intrinsic factors involved in understanding these complex phenomena include interactions with inner ear structures (cochlea, semicircular canals, utricle, and saccule) and their control systems (involvement of cortical areas, ascending and descending brainstem pathways, postural and motor reflexes, sensory and motor feedback mechanisms, multi-sensory-motor interactions, and integration), as proposed in the model of Zehr and Duysens (2004).

In considering all the factors involved, we advocate for the inclusion of quantifying gait parameters in medical, audiological, and physical therapy examinations. The rationale is justified because testing is simple, non-invasive, and safe. Moreover, it has positive predictive value in detecting incipient neurological disease, cognitive decline, dementia, and mortality (e.g., Basford et al., 2003; Moon et al., 2016; Zhou et al., 2017; Osoba et al., 2019; Szeto et al., 2021; Herssens et al., 2022). On this basis, we also advocate educating the lay public on this general information since it may help mitigate conditions, such as the risk of falling.

## Data availability statement

The raw data supporting the conclusions of this article will be made available by the authors, without undue reservation.

## Ethics statement

The studies involving human participants were reviewed and approved by the IRB Wayne State University. The patients/participants provided their written informed consent to participate in this study. Written informed consent was obtained from the individual(s) for the publication of any identifiable images or data included in this article.

## Author contributions

HB performed the data collection, contributed to the data analysis by performing the initial descriptive statistics, and wrote the initial draft of the manuscript. AC designed the research, analyzed the data including performing more detailed statistical analyses, constructed all figures and graphs, and reviewed and embellished the initial draft of the manuscript. Both authors contributed to the manuscript and approved the submitted version.

## Footnotes

1 The Nobel Prize in Physiology or Medicine (2022) was awarded to Svante Pääbo for the fundamental discovery that “gene transfer” had occurred from hominins to *Homo sapiens* following their migration out of Africa over 70,000 years ago. The most important, intriguing, and exceptional aspect of this work was performed by sequencing the entire Neanderthal genome, resulting in a new scientific discipline, termed “paleogenomics.”

2 Units of measurement associated with these normalization procedures include velocity (m)^s–1^; step length in meters (m), height or leg length (m); acceleration due to gravity (9.81 m^s–1^) g: and distance (D).
